# Usefulness of YouTube in Sharing Information about New Gene Therapy for Spinal Muscular Atrophy: A Content Analysis

**DOI:** 10.3390/healthcare11010147

**Published:** 2023-01-03

**Authors:** Kyeong Yeol Kim, Chan Woong Jang, Seok Young Chung, Myungsang Kim, Sung-Rae Cho, Han Eol Cho

**Affiliations:** 1 Department of Rehabilitation Medicine, Gangnam Severance Hospital, Yonsei University College of Medicine, Seoul 06273, Republic of Korea; 2 Rehabilitation Institute of Neuromuscular Disease, Yonsei University College of Medicine, Seoul 03722, Republic of Korea; 3 Department and Research Institute of Rehabilitation Medicine, Yonsei University College of Medicine, Seoul 03722, Republic of Korea; 4 Brain Korea 21 PLUS Project for Medical Science, Yonsei University College of Medicine, Seoul 03722, Republic of Korea; 5 Graduate Program of NanoScience and Technology, Yonsei University College of Medicine, Seoul 03722, Republic of Korea

**Keywords:** social media, YouTube, muscular atrophy, spinal, genetic therapy, education, distance, health education

## Abstract

This study aimed to objectively assess YouTube videos’ quality, reliability, and information delivery capability regarding novel spinal muscular atrophy treatments. Using the keywords “nusinersen”, “spinraza”, “ridisplam”, “evrysdi”, “onasemnogene abeparvovec”, and “zolgensma”, we were able to retrieve and screen 360 videos before settling on a final sample of 99 on 25 September 2022. Then, two independent raters used the mDISCERN and GQS instruments to evaluate the videos’ reliability and quality and the Information Delivery Capability (IDC) score to assess the videos’ accuracy and patient-friendliness. The quality, reliability, and information delivery capability of the videos about the new treatment for SMA were quite heterogeneous, with an average mDISCERN, GQS, and IDC score of 3.172 ± 0.899, 2.980 ± 1.025, and 4.141 ± 1.747, respectively. In-depth analysis showed that healthcare expert videos that explained contents while showing infographic supplements had good quality, reliability, and information delivery capability. As YouTube is already a dominant media platform, the public may obtain new information about novel therapeutics for SMA through YouTube. It is necessary to consider how SMA patients and caregivers can choose trusted sources with reliable information on YouTube, and our results can provide clues. Additionally, experts should strive to provide more accurate, reliable, and patient-oriented videos.

## 1. Introduction

Spinal muscular atrophy (SMA) is an autosomal recessive disorder caused by survival motor neuron (SMN) 1 gene dysfunction [1]. It is one of the most common inherited neuromuscular disorders and one of the most common fatal autosomal recessive disorders, with an incidence of 1 in 10,000 and a carrier frequency of 1 in 50 [2,3]. The SMN1 gene codes the SMN protein, essential for motor neuron survival in the spinal cord and brain stem [4]. This causes SMA patients to lose their muscle power and represents muscle hypotonia.

Recent advances in genetics have created a new paradigm for treating SMA. Nusinersen (Spinraza^®^; Biogen) was approved by the US Food and Drug Administration (FDA) in 2016, followed by onasemnogene abeparvovec (Zolgensma^®^; Novartis) in 2019 and risdiplam (Evrysdi^®^; Roche) in 2020 [5]. In humans, two forms of the SMN gene exist on each allele: a telomeric form (SMN1) and a centromeric form (SMN2). The SMN2 gene is identical to the SMN1 gene with the exception of a C-to-T substitution in an exonic splicing enhancer. Although most of the mRNA transcribed from the SMN2 gene is of the Δ7 form, which skips exon 7 via splicing, the full-length SMN2 mRNA containing exon 7 is produced at a rate of 5–10% of the total transcripts. Increased copy number of the SMN2 gene alleviates the severity of SMA [6]. Nusinersen and Risdiplam are the drugs increasing the production rate of intact SMN protein from the SMN2 gene through modulation of SMN2 splicing [7]. On the other hand, onasemnogene abeparvovec is a recombinant gene delivered to a patient’s DNA using a viral vector to produce SMN protein [8]. In other words, newly developed drugs modulate the disease course itself by targeting a deficiency of SMN protein. Since no specific treatment was previously available to modify the course of SMA, expectations for these therapies are tremendous [9].

Papers and conferences are important sources of information for medical professionals regarding these new treatments. However, it can be challenging for patients and their caregivers to acquire this information. Nowadays, it is clear that people are increasingly using the Internet to obtain medical information [10,11]. YouTube, the most famous video-sharing platform, also has become a source of medical information for patients [12]. Some previous studies have investigated whether health-related videos on YouTube are useful for teaching purposes [13]. However, there have been no studies about YouTube videos about new treatments on SMA patients. Although several videos about new gene therapies for SMA can be found on YouTube, it is unclear how accurate and patient-friendly this information is. Research is also required to determine how patients choose YouTube videos for SMA treatment.

Therefore, this study aimed to objectively assess the quality, reliability, and patient-friendliness of videos regarding novel SMA treatments (nusinersen, onasemnogene abeparvovec, and risdiplam) and to determine which YouTube videos best assist patients in comprehending novel SMA treatments.

## 2. Materials and Methods

### 2.1. Video Selection

Until 2022, the following three FDA-approved treatments for SMA had been released: nusinersen, onasemnogene abeparvovec, and risdiplam. We conducted a search on YouTube on 25 September 2022 using the keywords “nusinersen”, “spinraza”, “ridisplam”, “evrysdi”, “onasemnogene abeparvovec”, and “zolgensma”.

**Figure 1:**
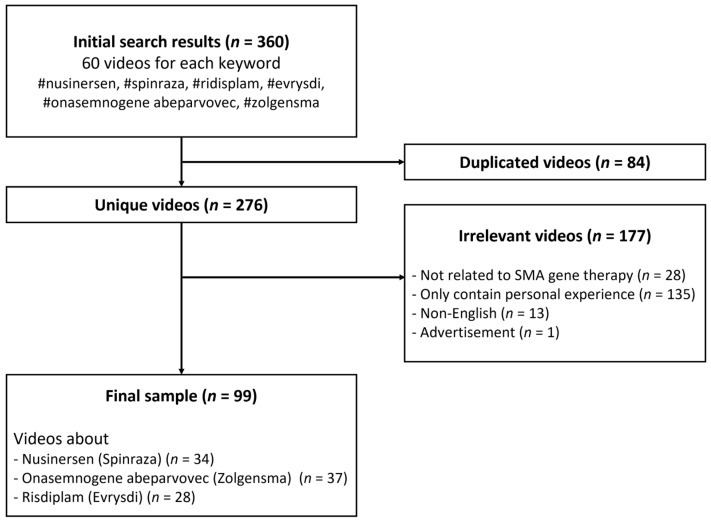
Flowchart of the search process for videos related to gene therapy for SMA.

A previous study showed that more than 90% of Internet users clicked on the first three pages of search results [14]; therefore, the top 60 videos for each keyword were listed at the end of the search. Then, we excluded videos that (1) were duplicated (*n* = 84), (2) were irrelevant or not directly related to SMA gene therapy (*n* = 28), (3) only contained personal experience (*n* = 135), (4) were in non-English languages (*n* = 13), and (5) were advertisements without information about medication (*n* = 1). Finally, 99 videos remained for data analysis (Figure 1). No ethics committee permission was necessary because there were no human subjects in this study.

### 2.2. Data Extraction and Processing

To define the properties of each video, its basic descriptive characteristics were collected on the day of the search. These included title, uploader, length of the video, upload date, and the total number of views, likes, and comments.

### 2.3. Video Subgroup According to the Expertise of the Lecturer and Educational Methods

**Figure 2:**
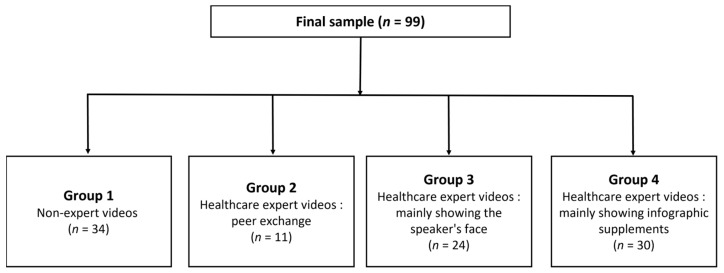
Subgroup of videos according to the expertise of the lecturer and educational methods.

Each video was divided into four categories according to the expertise of the lecturer and educational methods, as follows: “Group 1” nonexpert videos; “Group 2” healthcare expert videos—peer exchange; “Group 3” healthcare expert videos—mainly showing the speaker’s face; and “Group 4” healthcare expert videos—mainly showing infographic supplements (Figure 2).

### 2.4. Assessment Tools for Reliability, Quality

Video reliability was evaluated by the 5-point scale mDISCERN tool, which is adapted to YouTube videos and was adapted from the original DISCERN for the assessment of written health information by Charnock et al. [15]. The mDISCERN scale included five questions which are as follows: (1) “Are the aims clear and achieved?” (2) Are reliable sources of information used?” (3) “Is the information presented both balanced and unbiased?” (4) “Are additional sources of information listed for patient reference?” and (5) “Are areas of uncertainty mentioned?” Each of the five questions was scored on a two-point scale ranging from 0 to 1. A higher mDISCERN score indicated greater reliability. The maximum potential score was 5, with significance in the reliability when a mDISCERN score is 3 or greater [16].

The Global Quality Scale (GQS) developed by Bernart et al. was used to assess the overall quality of the video content [17]. It is a five-point scale that assesses flow, ease of video use, and video quality, and the points are described as follows: (1) signifies poor quality, poor flow, and most information is missing so that it is not helpful for patients; (2) signifies that the video is generally poor, with some information given but of limited use to patients; (3) means that it is of moderate quality, and some important information is adequately discussed; (4) signifies good quality, good flow, and most relevant information is covered, making it useful for patients, and (5) means excellent quality and excellent flow, making it very useful for patients. Scores of 1–2, 3, and 4–5 points indicate low, moderate, and high quality, respectively [18].

### 2.5. New Scores for In-Depth Analysis of Information Delivery Capability for SMA Gene Therapy

The SMA gene therapies that we are focusing on are recently developed medications. Therefore, we attempted to evaluate how accurate and patient-friendly drug information may be presented to patients with SMA. For this in-depth analysis, a new scoring system called the “Information Delivery Capability (IDC)” score was developed by the authors.

To develop the IDC score, we invited five specialist panels working in a hospital specializing in neuromuscular diseases and experiencing treatment-related counseling with SMA patients. We requested panels about what kind of questions they received from patients most frequently and what they thought the video should contain. Subsequently, the final IDC score was determined through a panel discussion. The IDC contains the following seven items: (1) Is the treatment mechanism described? (2) Did the video clarify the effects of treatment? (3) Are safety-related details disclosed? (4) Do videos use evidence-based data? (5) Did the videos cover how to use medication (drug administration route, administration cycle, and dose)? (6) Did the video employ visual aids to assist viewers in their understanding? (7) Did the video employ terminology that the average person could understand?

In the case of item 5, 2 points were awarded for all explanations of the drug administration route, administration cycle, and dose, 1 point for any explanation, and 0 points for no explanation. For the other items, 1 point was awarded if an explanation was provided and 0 points if not. If it contained even one inaccurate explanation or action, it received 0 points. IDC is based on the sum of the points from the individual domains. The highest possible score for the video was 8 points, and the lowest score was 0. The higher the score, the greater the information delivery capability of the video.

The inter-rater reliability of the IDC score was checked before the study because the IDC item was newly developed. Two independent raters scored 50 other sample videos on YouTube and were blinded to each other’s responses. The Cohen kappa inter-rater reliability was 0.938 (*p* < 0.001), indicating an almost perfect agreement [19].

### 2.6. Data Processing and Assessment

Two independent reviewers who specialize in neuromuscular disorders evaluated each video using mDISCERN, GQS, and IDC after training to analyze the video in the same manner. The content and information of each video were reviewed. Discrepancies in the scores for the same video between reviewers were resolved by consensus until an agreement was reached.

### 2.7. Statistical Analysis

Descriptive data are presented as median (interquartile range) for days since the videos’ upload, number of views, number of likes, number of comments, duration (seconds), and mean ± standard deviation for the mDISCERN, GQS, and IDC. The Shapiro–Wilk test was applied to approximate the normality of the data. For analysis by each therapy and video category, ANOVA with Scheffe’s posthoc test was used to compare the mDISCERN score, GQS, and IDC score, and the Kruskal–Wallis test and the Mann–Whitney test as posthoc tests were used for days since the videos’ upload, number of views, number of likes, number of comments, and duration. Inter-rater reliability was measured separately for the scoring of the mDISCERN, GQS, and IDC using Cohen’s weighted kappa coefficient, with significance set at *P* > 0.6. All analyses were performed using RStudio software (R version 4.1.2). Statistical significance was set at *P* < 0.05 for parameters other than inter-rater reliability.

## 3. Results

### 3.1. Basic Characteristics

Table 1 presents the baseline characteristics of the videos. The median number of days since the videos’ upload was 775 (526–1171 days). The median numbers of views, likes, and comments were 298 (164.5–1,138), 6.5 (0–21.5), and 0 (0–2.5), respectively. The median video length was 335 s (227–486 s). To assess reliability, quality, and information delivery capabilities, the average scores of the mDISCERN, GQS, and IDC were 3.172 ± 0.899, 2.980 ± 1.025, and 4.141 ± 1.747, respectively.

The kappa scores indicated good agreement between the raters, showing that the inter-rater reliabilities for the GQS, DISCERN, and IDC were 0.824, 0.796, and 0.903, respectively.

### 3.2. Differences in Gene Therapies

**Figure 3:**
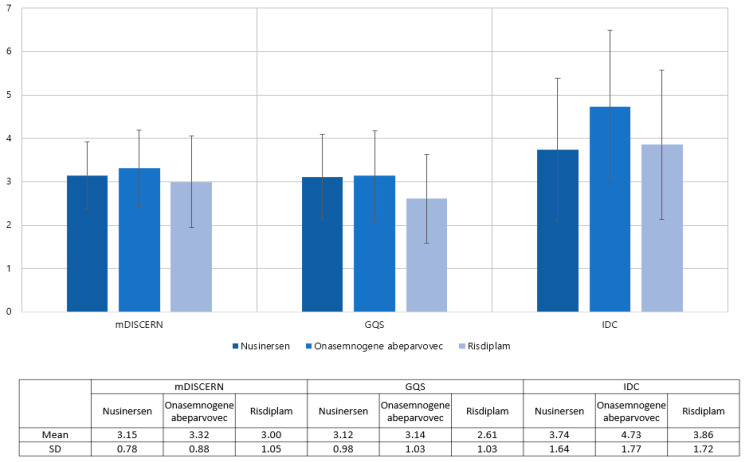
Distribution of mDISCERN, GQS, and IDC across the type of medications.

The analysis includes 34 nusinersen, 37 onasemnogene abeparvovec, and 28 Risdiplam videos out of the 99 evaluated videos (Table 2). Nusinersen’s mean mDISCERN score was 3.15, onasemnogene abeparvovec’s was 3.32, and risdiplam’s was 3.00. For nusinersen, onasemnogene abeparvovec, and risdiplam, the mean GQS values were 3.12, 3.14, and 2.61, respectively. There was no difference between the groups in terms of the mDISCERN (*p* = 0.355) and GQS (*p* = 0.076). The IDC score was checked as 3.74 for nusinersen, 4.73 for onasemnogene abeparvovec, and 3.86 for risdiplam. ANOVA showed differences among groups (*p* = 0.033); however, the posthoc comparison did not reveal differences between any of the groups (Figure 3).

### 3.3. Comparison of Differences by a Subgroup of Videos

There were 11 videos in Group 1, 34 videos in Group 2, 24 videos in Group 3, and 30 videos in Group 4. The mean GQS was 2.47, 2.45, 2.67, and 4.00; the mean mDISCERN score was 3.50, 3.91, 3.38, and 5.57; and the mean IDC total scores were 3.50, 3.91, 3.38, and 5.57, respectively (Table 3).

**Figure 4:**
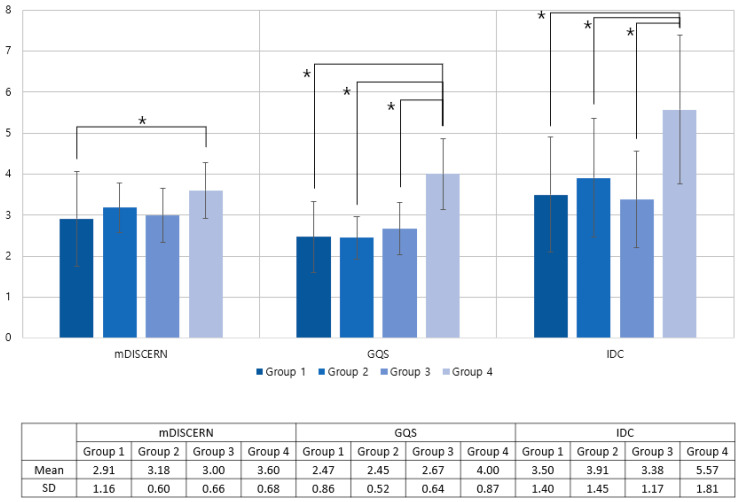
Distribution of mDISCERN, GQS, and IDC as a group by the expertise of lecturer and educational methods. Group 1: nonexpert videos; Group 2: healthcare expert videos—peer exchange; Group 3:healthcare expert videos—mainly showing the speaker’s face; Group 4: healthcare expert videos—mainly showing infographic supplements. GQS: Global Quality Score; IDC: Information Delivery Capability. * Significant difference.

The results of the ANOVA test showed significant differences in the mDISCERN score (*p* = 0.013), GQS score (p<0.001), and IDC (*p* < 0.001) among the subgroups. In the posthoc analysis, Group 4 showed significantly higher scores than did the other groups. Group 4 showed a higher GQS than Group 1 (*p* < 0.001), Group 2 (*p* < 0.001), and Group 3 (*p* < 0.001). For mDISCERN, the Group 4 score was higher than that of Group 1(*p* = 0.023). For IDC, Group 4 showed a significantly higher score than Group 1 (*p* < 0.001), Group 2 (*p* = 0.024), and Group 3 (*p* < 0.001). There were no significant differences among the other groups (Figure 4).

## 4. Discussion

Genetic illnesses such as SMA have entered a new therapeutic paradigm as a result of the spectacular advancement of genetics, starting with the release of nursinersen in 2017, onasemnogene abeparvovec in 2019, and ridisplam in 2020. A new treatment that potentially provides a fundamental cure has raised patients’ expectations, leading to a request for information on these novel medications. Currently, the public is increasingly obtaining information through the Internet, and YouTube, a leading video platform, is gaining popularity as a means of searching for new information. It is implicit that assessing whether innovative treatment information about SMA is presented accurately and patiently on YouTube is critical.

The US FDA approved three drugs, each with somewhat different specifics. Nusinersen is an antisense oligonucleotide drug and alters SMN2 pre-mRNA splicing to encourage increased production of the full-length SMN protein [20]. As it cannot cross the blood–brain barrier, it should be administered intrathecally. Onasemnogene abeparvovec is an adeno-associated viral (AAV9) vector containing a copy of the SMN1 gene. It can be administered through a vein, and then AAV9 vectors insert the SMN1 gene into the patient’s DNA to induce SMN protein production [21]. Risdiplam is an SMN2 mRNA splicing modifier, similar to nusinersen. However, its small molecular size allows it to cross the blood–brain barrier, so patients take medication orally [22].

Previous studies have shown that YouTube has great influence as a source of health-related information. For example, COVID-19 vaccine-related videos on YouTube were viewed over 30 million times globally. Previous studies showed that YouTube is not a reliable source of medical and health-related information [23]. Nonetheless, we expected that the quality of medical knowledge would be good for specialized treatment for rare diseases because that could be mainly provided by medical professionals.

Unfortunately, we found that the quality and reliability of the videos about the new treatment for SMA were quite heterogeneous, with average scores of 3.172 on the mDISCERN and 2.980 on the GQS. These results are not very different from the results of previous studies on YouTube as a source of medical information [23,24,25,26,27]. Our results show that YouTube is not a good tool for delivering medical information regardless of disease rarity. It may be because there is no peer-review system. Interestingly, some videos showed exceptionally high scores. These results suggest that it is necessary to consider how patients can select videos that deliver accurate, reliable, and patient-friendly information.

The influence of YouTube is already enormous, and we cannot entirely monitor all the videos. It is unavoidable that patients and caregivers must select appropriate videos with reliable information. We attempted to identify what factors are important in selecting reliable videos.

Our study found that the reliability, quality, and informational delivery capability of the videos did not differ according to the type of medication used. In addition, there was no difference in the number of views, likes, comments, and video length. The only difference was observed in the posting dates, likely because nusinersen has the longest posting intervals since its early introduction.

We found a difference in the results according to the expertise of the lecturer and instructional approaches. ‘Healthcare expert videos—mainly show infographic supplements’ scored significantly better than the other groups. The group average mDISCERN score was 3.6, the GQS score was 4.0, indicating good quality, and the IDC score was 5.57 out of 7, showing that information was explained in a patient-friendly manner. This was consistent with earlier research indicating a difference in the quality and reliability of videos based on healthcare professions versus nonprofessions [28,29,30]. Interestingly, even among expert videos, ‘healthcare expert videos—mainly showing infographic supplements’ also scored better than the other groups. Peer exchange videos showed low scores because they are likely to target medical professions and are not patient-centered. The difference in scores according to infographic supplements may be due to the difference in the will to deliver information; infographic supplements are supposed to explain basic knowledge to the audience step-by-step [31].

In addition, we would like to discuss the IDC scoring system we created. Existing instruments, such as the mDISCERN and the GQS, which were not originally created for video assessment, are nonetheless widely utilized for evaluating videos with health-related information [12]. In fact, these scores are not adequate to make a sufficient assessment of the health-related information. We developed the IDC score in consultation with medical specialists. This score represents the basic necessities of health-related videos with SMA gene therapy. Through the development, validation, and modification of scores like ours, we believed that medical professionals would set the standard for producing accurate and reliable videos. We think that it will be very helpful in delivering accurate and reliable health-related information if professionals undertake efforts such as making a good video evaluation tool through exchange of opinions and posting high-scoring videos to patient and caregivers [32].

### Strengths and Limitations

The strength of this study is that we conducted YouTube research on cutting-edge, novel, and paradigm-shifting treatments for a rare and incurable genetic disease. Through this study, it is possible to estimate whether patients can obtain proper information about new treatments on YouTube.

However, this study had inherent limitations. First, there may be some debate over whether there are enough YouTube videos to analyze since new medicines have only been released for around three years after US FDA approval. However, we think it is also important to analyze YouTube videos at the early stages after drug release, when patients are most interested. Second, we did not include all videos with new SMA treatment. However, a previous study showed that more than 90% of Internet users clicked on the first three pages of search results [18]; therefore, our sample size is sufficient for the study. Third, there were only few comments shown in included videos. This is probably because the disease’s specificity restricted the number of viewers. Last, we could not evaluate videos in other languages that may represent the entire population of YouTube videos, because we only analyzed English-language videos.

## 5. Conclusions

YouTube videos’ quality, reliability, and information delivery capability, which provide information about new treatments for SMA, were heterogeneous. In-depth analysis showed that ‘Healthcare expert videos’—mainly infographic supplements—had good quality, reliability, and information delivery capability. As YouTube is already a dominant media platform, there is a high possibility that the public will obtain new information about novel therapeutics through YouTube. It is necessary to consider how the public can choose good videos, and this study may serve as a springboard for future research. Since our study found that infographic supplements used by medical professionals in videos are highly informative, more research will be required to determine whether this tendency exists in other YouTube videos.

## Figures and Tables

**Table 1:** Basic characteristics of included videos (*n* = 99).

Characteristics	Median (IQR)
Days since the videos’ upload	775 (526–1171)
Number of views	298 (164.5–1138)
Number of likes	6.5 (0–21.5)
Number of comments	0 (0–2.5)
Duration (seconds)	335 (227–486)
Scoring	Mean ± SD
mDISCERN, total	3.172 ± 0.899
mDISCERN, item 1	0.949 ± 0.219
mDISCERN, item 2	0.869 ± 0.338
mDISCERN, item 3	0.828 ± 0.377
mDISCERN, item 4	0.333 ± 0.471
mDISCERN, item 5	0.192 ± 0.394
Global Quality Score	2.980 ± 1.025
Information Delivery Capability	4.141 ± 1.747
(1) Is the treatment’s mechanism described?	0.505 ± 0.500
(2) Did the video clarify the treatment’s effect?	0.838 ± 0.368
(3) Are safety-related details disclosed?	0.323 ± 0.468
(4) Did videos use evidence-based data?	0.576 ± 0.494
(5) Did videos cover how to use medication?	0.899 ± 0.718
(6) Did the video employ visual aids to assist viewers in understanding?	0.343 ± 0.475
(7) Did the video employ terminology that the average person could understand?	0.657 ± 0.475

**Table 2:** Characteristics of the videos according to SMA treatment.

Characteristics, Median (IQR)	Medications			*p*-Value
	Nusinersen	Onasemnogene Abeparvovec	Risdiplam	
Days since the videos upload	1075 (697.25–1692.50)	625 (462–1113)	764.5 (526–976)	0.002
Number of views	223 (142–2879.25)	374 (226–1174)	350.5 (105.5–777)	0.393
Number of likes	6.5 (0–25)	8.5 (2.5–19)	3 (0–17.75)	0.331
Number of comments	0 (0–7.75)	0 (0–5)	0 (0–0)	0.103
Duration (s)	302 (222–483)	381 (244–462)	395 (214–532)	0.796
Scoring, mean ± SD				
mDISCERN, total	3.15 ± 0.78	3.32 ± 0.88	3.00 ± 1.05	0.355
mDISCERN, item 1	0.94 ± 0.24	1 ± 0	0.89 ± 0.31	
mDISCERN, item 2	0.94 ± 0.24	0.86 ± 0.35	0.79 ± 0.42	
mDISCERN, item 3	0.85 ± 0.36	0.86 ± 0.35	0.75 ± 0.44	
mDISCERN, item 4	0.26 ± 0.45	0.41 ± 0.50	0.32 ± 0.48	
mDISCERN, item 5	0.15 ± 0.36	0.19 ± 0.40	0.25 ± 0.44	
Global Quality Score	3.12 ± 0.98	3.14 ± 1.03	2.61 ± 1.03	0.076
Information Delivery Capability	3.74 ± 1.64	4.73 ± 1.77	3.86 ± 1.72	0.033
(1) Is the treatment’s mechanism described?	0.44 ± 0.50	0.65 ± 0.48	0.39 ± 0.50	
(2) Did the video clarify the treatment’s effect?	0.82 ± 0.39	0.95 ± 0.23	0.71 ± 0.46	
(3) Are safety-related details disclosed?	0.24 ± 0.43	0.43 ± 0.50	0.29 ± 0.46	
(4) Did videos use evidence-based data?	0.59 ± 0.50	0.54 ± 0.51	0.61 ± 0.50	
(5) Did videos cover how to use medication?	0.65 ± 0.65	1.11 ± 0.77	0.93 ± 0.66	
(6) Did the video employ visual aids to assist viewers in understanding?	0.38 ± 0.49	0.41 ± 0.50	0.21 ± 0.42	
(7) Did the video employ terminology that the average person could understand?	0.62 ± 0.49	0.65 ± 0.48	0.71 ± 0.46	

**Table 3:** Characteristics of the videos according to a subgroup (expertise of lecturer and educational methods).

Characteristics, Median (IQR)	Video Classification				*p* Value
	Group 1 (*n* = 34)	Group 2 (*n* = 11)	Group 3 (*n* = 24)	Group 4 (*n* = 30)	
Days since the videos’ upload	976 (591.5–1214.75)	817 (801–1170.5)	717.5 (522.75–1075)	598 (446.25–876.25)	0.091
Number of views	256 (150–798.75)	427 (281–849)	247.5 (125.75–681)	820 (174.5–7377.5)	0.102
Number of likes	15 (9–24)	6 (2–8.5)	0 (0–5)	3.5 (0–71.5)	<0.001
Number of comments	0 (0–875)	0 (0–2.5)	0 (0–0)	0 (0–2)	0.147
Duration (seconds)	435 (324–486)	280 (248–433)	219 (166–272)	422 (216–787)	<0.001
Scoring, mean ± SD					
mDISCERN, total	2.91 ± 1.16	3.18 ± 0.60	3.00 ± 0.66	3.60 ± 0.68	0.013
mDISCERN, item 1	0.91 ± 0.29	0.91 ± 0.30	0.96 ± 0.20	1.00	
mDISCERN, item 2	0.65 ± 0.49	1.00	0.96 ± 0.20	1.00	
mDISCERN, item 3	0.59 ± 0.50	1.00	0.88 ± 0.34	1.00	
mDISCERN, item 4	0.56 ± 0.50	1.00	0.04 ± 0.20	0.43 ± 0.50	
mDISCERN, item 5	0.21 ± 0.41	0.27 ± 0.47	0.17 ± 0.38	0.17 ± 0.38	
Global Quality Score	2.47 ± 0.86	2.45 ± 0.52	2.67 ± 0.64	4.00 ± 0.87	<0.001
Information Delivery Capability	3.50 ± 1.40	3.91 ± 1.45	3.38 ± 1.17	5.57 ± 1.81	<0.001
(1) Is the treatment’s mechanism described?	0.35 ± 0.49	0.55 ± 0.52	0.33 ± 0.48	0.80 ± 0.41	
(2) Did the video clarify the treatment’s effect?	0.74 ± 0.45	1	0.92 ± 0.28	0.83 ± 0.38	
(3) Are safety-related details disclosed?	0.21 ± 0.41	0.45 ± 0.52	0.17 ± 0.38	0.53 ± 0.51	
(4) Did videos use evidence-based data?	0.38 ± 0.49	1	0.58 ± 0.50	0.63 ± 0.49	
(5) Did videos cover how to use medication?	0.74 ± 0.57	0.82 ± 0.60	0.63 ± 0.65	1.33 ± 0.80	
(6) Did the video employ visual aids to assist viewers in understanding?	0.12 ± 0.33	0	0	1	
(7) Did the video employ terminology that the average person could understand?	0.97 ± 0.17	0.09 ± 0.30	0.75 ± 0.44	0.43 ± 0.50	

Group 1: nonexpert videos; Group 2: healthcare expert videos—peer exchange; Group 3: healthcare expert videos—mainly showing the speaker’s face; Group 4: healthcare expert videos—mainly showing infographic supplements.

## Data Availability

Data are available on request owing to restrictions. The data presented in this study are available upon request from the corresponding author.

## References

[1] Lunn M.R., Wang C.H. (2008). Spinal muscular atrophy. Lancet.

[2] Ogino S., Leonard D.G.B., Rennert H., Ewens W.J., Wilson R.B. (2002). Genetic risk assessment in carrier testing for spinal muscular atrophy. Am. J. Med. Genet..

[3] Prior T.W., Snyder P.J., Rink B.D., Pearl D.K., Pyatt R.E., Mihal D.C., Conlan T., Schmalz B., Montgomery L., Ziegler K. (2010). Newborn and carrier screening for spinal muscular atrophy. Am. J. Med. Genet. Part A.

[4] Fan L., Simard L.R. (2002). Survival motor neuron (SMN) protein: Role in neurite outgrowth and neuromuscular maturation during neuronal differentiation and development. Hum. Mol. Genet..

[5] Gandhi G., Abdullah S., Foead A.I., Yeo W.W.Y. (2021). The potential role of miRNA therapies in spinal muscle atrophy. J. Neurol. Sci..

[6] Kolb S.J., Kissel J.T. (2015). Spinal muscular atrophy. Neurol. Clin..

[7] Li Q. (2020). Nusinersen as a therapeutic agent for spinal muscular atrophy. Yonsei Med. J..

[8] Nicolau S., Waldrop M.A., Connolly A.M., Mendell J.R. (2021). Spinal muscular atrophy. Semin. Pediatr. Neurol..

[9] Gusset N., Stalens C., Stumpe E., Klouvi L., Mejat A., Ouillade M.-C., de Lemus M. (2021). Understanding European patient expectations towards current therapeutic development in spinal muscular atrophy. Neuromuscul. Disord..

[10] Rice R.E. (2006). Influences, usage, and outcomes of internet health information searching: Multivariate results from the pew surveys. Int. J. Med. Inform..

[11] Rozenblum R., Bates D.W. (2013). Patient-centred healthcare, social media and the internet: The perfect storm?. BMJ Qual. Saf..

[12] Drozd B., Couvillon E., Suarez A. (2018). Medical Youtube videos and methods of evaluation: Literature review. JMIR Med. Educ..

[13] Jang C.W., Bang M., Park J.H., Cho H.E. (2022). Value of online videos as a shoulder injection training tool for physicians and usability of current video evaluation tools. Int. J. Environ. Res. Public Health.

[14] Tolu S., Yurdakul O.V., Basaran B., Rezvani A. (2018). English-language videos on Youtube as a source of information on self-administer subcutaneous anti-tumour necrosis factor agent injections. Rheumatol. Int..

[15] Charnock D., Shepperd S., Needham G., Gann R. (1999). Discern: An instrument for judging the quality of written consumer health information on treatment choices. J. Epidemiol. Community Health.

[16] Langford B., Hooten W.M., D’Souza S., Moeschler S., D’Souza R.S. (2021). Youtube as a source of medical information about spinal cord stimulation. Neuromodulation.

[17] Bernard A., Langille M., Hughes S., Rose C., Leddin D., Van Zanten S.V. (2007). A systematic review of patient inflammatory bowel disease information resources on the world wide web. Off. J. Am. Coll. Gastroenterol..

[18] Kocyigit B.F., Nacitarhan V., Koca T.T., Berk E. (2019). Youtube as a source of patient information for ankylosing spondylitis exercises. Clin. Rheumatol..

[19] Gisev N., Bell J.S., Chen T.F. (2013). Interrater agreement and interrater reliability: Key concepts, approaches, and applications. Res. Soc. Adm. Pharm..

[20] Hua Y., Sahashi K., Hung G., Rigo F., Passini M.A., Bennett C.F., Krainer A.R. (2010). Antisense correction of SMN2 splicing in the cns rescues necrosis in a type III SMA mouse model. Genes Dev.

[21] McMillan H.J., Proud C.M., Farrar M.A., Alexander I.E., Muntoni F., Servais L. (2022). Onasemnogene abeparvovec for the treatment of spinal muscular atrophy. Expert Opin. Biol. Ther..

[22] Ratni H., Ebeling M., Baird J., Bendels S., Bylund J., Chen K.S., Denk N., Feng Z., Green L., Guerard M. (2018). Discovery of risdiplam, a selective survival of motor neuron-2 (SMN2) gene splicing modifier for the treatment of spinal muscular atrophy (SMA). J. Med. Chem..

[23] Osman W., Mohamed F., Elhassan M., Shoufan A. (2022). Is youtube a reliable source of health-related information? A systematic review. BMC Med. Educ..

[24] Jang C.W., Kim M., Kang S.-W., Cho H.E. (2022). Reliability, quality, and educational suitability of Tiktok videos as a source of information about scoliosis exercises: A cross-sectional study. Healthcare.

[25] Chang M.C., Park D. (2021). Youtube as a source of information on epidural steroid injection. J Pain Res.

[26] Li H.O.-Y., Bailey A., Huynh D., Chan J. (2020). Youtube as a source of information on COVID-19: A pandemic of misinformation?. BMJ Glob. Health.

[27] Yildiz M.B., Yildiz E., Balci S., Özçelik Köse A. (2021). Evaluation of the quality, reliability, and educational content of Youtube videos as an information source for soft contact lenses. Eye Contact Lens.

[28] Şahin A., Şahin M., Türkcü F.M. (2019). Youtube as a source of information in retinopathy of prematurity. Ir. J. Med. Sci..

[29] Onder M.E., Zengin O. (2021). Youtube as a source of information on gout: A quality analysis. Rheumatol. Int..

[30] Pathak R., Poudel D., Karmacharya P., Pathak A., Aryal M., Mahmood M., Donato A. (2015). Youtube as a source of information on Ebola virus disease. North Am. J. Med. Sci..

[31] Salama A., Panoch J., Bandali E., Carroll A., Wiehe S., Downs S., Cain M.P., Frankel R., Chan K.H. (2020). Consulting “Dr. Youtube”: An objective evaluation of hypospadias videos on a popular video-sharing website. J. Pediatr. Urol..

[32] Laversin S., Baujard V., Gaudinat A., Simonet M.A., Boyer C. (2011). Improving the transparency of health information found on the internet through the HONcode: A comparative study. Stud. Health Technol. Inform..

